# Prevalence of anxiety symptoms and associated factors at 2 months postpartum, results from a 2021 French national prospective cohort study

**DOI:** 10.1192/j.eurpsy.2024.1799

**Published:** 2024-12-27

**Authors:** Alexandra Doncarli, Virginie Demiguel, Camille Le Ray, Catherine Deneux-Tharaux, Elodie Lebreton, Gisèle Apter, Julie Boudet-Berquier, Catherine Crenn-Hebert, Marie-Noëlle Vacheron, Nolwenn Regnault, Sarah Tebeka, Members of the ENP2021 Study Group

**Affiliations:** 1 Santé publique France, the national public health agency, Saint-Maurice, France; 2 Université Paris Cité, Obstetrical Perinatal and Pediatric Epidemiology Research Team (EPOPé), CRESS U1153, INSERM, INRAE; Port Royal Maternity Unit, Cochin Hospital, Assistance Publique-Hôpitaux de Paris, Université Paris Cité, FHU Prema, Paris, France; 3 Université Paris Cité, Obstetrical Perinatal and Pediatric Epidemiology Research Team (EPOPé), CRESS U1153, INSERM, INRAE, Paris, France; 4 Le Havre Hospital, Perinatal and Child Psychiatry; Normandie University, Le Havre, France; 5 Louis-Mourier Hospital, Department of Gynecology and Obstetrics, Assistance Publique-Hôpitaux de Paris, Colombes;Regional Health Agency of Ile de France (ARS-IDF), Paris, France; 6 Sainte Anne Hospital, GHU Paris Psychiatrie et Neurosciences, Consultation d’Information, de Conseils et d’Orientation des femmes suivies pour troubles psychiques, enceintes, ou avec désir d’enfant (CICO), Paris, France

**Keywords:** Maternal anxiety, postpartum, depression, risk factors

## Abstract

**Background:**

Postpartum anxiety (PPA) symptoms have harmful effects on child development and mother–infant interactions. Accordingly, in-depth knowledge of associated risk factors is crucial for prevention policies. This study aimed to estimate PPA symptom prevalence at 2 months and to identify associated risk factors in a representative sample of all women who gave birth in France in 2021, and in two subgroups: women with no postpartum depression (PPD) symptoms, and those with no history of mental health care.

**Methods:**

Among the 12,723 women included in the representative French national perinatal survey 2021ENP, 7,133 completed the Edinburgh Postnatal Depression Scale (EPDS) self-administered questionnaire – including three anxiety-specific items (EPDS-3A) – at 2 months postpartum. We estimated the adjusted prevalence ratios (aPR) of PPA symptoms using Poisson regression models with robust variance.

**Results:**

PPA symptom prevalence at 2 months was 27.6% (95% CI [26.5–28.8]). Associated risk factors were: age ≤ 34 years (maximum aPR = 1.38 [1.22–1.58] obtained for persons aged 25–29 years vs. 35–39 years), poorer health literacy (1.15 [1.07–1.23]), a history of medical termination of pregnancy (1.32 [1.05–1.68]), psychological (1.31 [1.17–1.47]) or psychiatric (1.42 [1.24–1.63]) care history since adolescence, nulliparity (1.23 [1.12–1.35]), no weight gain or loss (1.29 [1.03–1.61] vs. 9–15 kg gain) or gain ≥23 kg (1.20 [1.00–1.43]) during pregnancy, ≥3 pregnancy-related emergency consultations (1.16 [1.03–1.31] vs. none), poor/good support during pregnancy, (1.16 [1.00–1.34] and 1.15 [1.05–1.26], respectively, vs. very good), sadness (1.52 [1.36–1.69]), anhedonia (1.48 [1.27–1.72]), or both (1.99 [1.79–2.21]) during pregnancy, not at all/not very satisfied with pain management during childbirth (1.16 [1.01–1.32] vs. quite/very satisfied). Similar risk factors were found in the ‘no PPD symptoms’ and ‘no history of mental health care’ subgroups.

**Conclusions:**

Estimated PPA symptom prevalence at 2 months in our study sample was 27.6%. The risk factors we identified may guide future prevention policies.

## Introduction

Although less studied than postpartum depression (PPD), postpartum anxiety (PPA) is an important feature of postpartum psychological suffering, whether it manifests alone or in comorbidity with PPD [1]. Some authors have even observed a higher prevalence of anxiety symptoms than depressive symptoms in the perinatal period [2]. A meta-analysis conducted by Dennis et al. in 2017, which included 22 studies of women from high-resource countries (*n* = 19,158), found a pooled self-reported PPA (i.e., 5–12 weeks) symptom prevalence of 14.9% (95% CI [12.3–17.5]) [3].

PPA disorders are associated with psychological distress and reduced self-esteem [4]. PPA symptoms impact child development and are associated with negative mother–infant interactions [5]. More specifically, they are associated with poorer overall social–emotional development of the child, externalizing and internalizing behavioral problems, temperamental negative emotionality, poorer language capabilities in infancy, and weaker gross motor skills [6]. Moreover, children with a parent presenting PPA symptoms have a higher risk of developing anxiety [7, 8].

All these findings highlight the need for early identification of PPA symptoms in mothers and the importance of appropriate psychological and/or psychiatric support. With regard to early identification, it is especially important to have a thorough understanding of the associated risk factors of these symptoms [5]. To date, only a small number of studies have focused on risk factors using multivariable analyses [2, 9–15]. Almost all had small sample sizes and were not representative at the national level. Instead, several bivariable studies have been performed. These show that, just as is the case for PPD, the emergence of PPA symptoms is multifactorial and is more frequent in persons with a history of mental health problems [15, 16].

Most studies investigate either depression or anxiety and do not take into account the fact that both coexist in a large proportion of the concerned women, whether in the general population [17], in pregnant women [18], and women at 2 months postpartum [19]. To our knowledge, only one study to date has investigated women with PPA symptoms who do not present PPD symptoms. Specifically, it comprised 4,451 women who delivered in 2009–2010 in the United States [2].

The representative 2021 French national population-based perinatal survey (2021 ENP) provided us with the opportunity to study PPA symptoms at 2 months in the French context. Unlike previous ENP surveys, the 2021 survey included the Edinburgh Postnatal Depression Scale (EPDS). PPA symptoms were detected in the whole sample and two subgroups using the EPDS’s dedicated three-item sub-scale (EPDS-3A). The two subgroups were (i) women with no PPD symptoms (defined as an EPDS score < 13) and (ii) those with no history of mental health care since adolescence.

The present study, which used data from 2021 ENP, aimed to (i) estimate the prevalence of PPA symptoms at 2 months, and (ii) study the association between participant characteristics and PPA symptoms. Results are presented for the whole study population and two abovementioned subgroups.

## Methods

### Survey methodology and data collected

All women aged 15 years and over who gave birth in France in the same week of March 2021 after at least 22 weeks of gestation to at least one live newborn weighing a minimum 500 g were invited to participate in 2021 ENP [20]. A total of 12,723 women comprised this national representative sample; the overall coverage rate was 99% (i.e., public/private maternity hospitals and birth centers combined) [20]. The inference to all women who gave birth in France in 2021 has been detailed in recent previous publications [20, 21].

The 2021 ENP comprised a face-to-face interview with specially trained midwives during mothers’ postpartum stay in the maternity ward and the completion of a 2-month postpartum questionnaire by telephone or Internet. Of the 10,958 women who agreed to the maternity ward-based interview, 7,399 (67.5%) also completed the questionnaire. The maternity ward interview explored demographic and socioeconomic characteristics, various behaviors before and during pregnancy (e.g., tobacco consumption, pregnancy-related emergency visits, walk-in consultations), and medical surveillance during the prenatal period. The midwives also collected data from patients’ medical files on their maternal history, the course of their pregnancy, their delivery (e.g., pain management, delivery method), and the health status of their child at birth. The 2-month postpartum questionnaire collected information about mothers’ experience of pregnancy and childbirth, the organization of their return home, their health (including an assessment of their mental health by completing the EPDS [see above]), and their child’s health.

The maternity ward interview and 2-month postpartum questionnaire are available in appendices 3 and 4 of the 2021 ENP report [21].

### Participants

**Figure 1.**
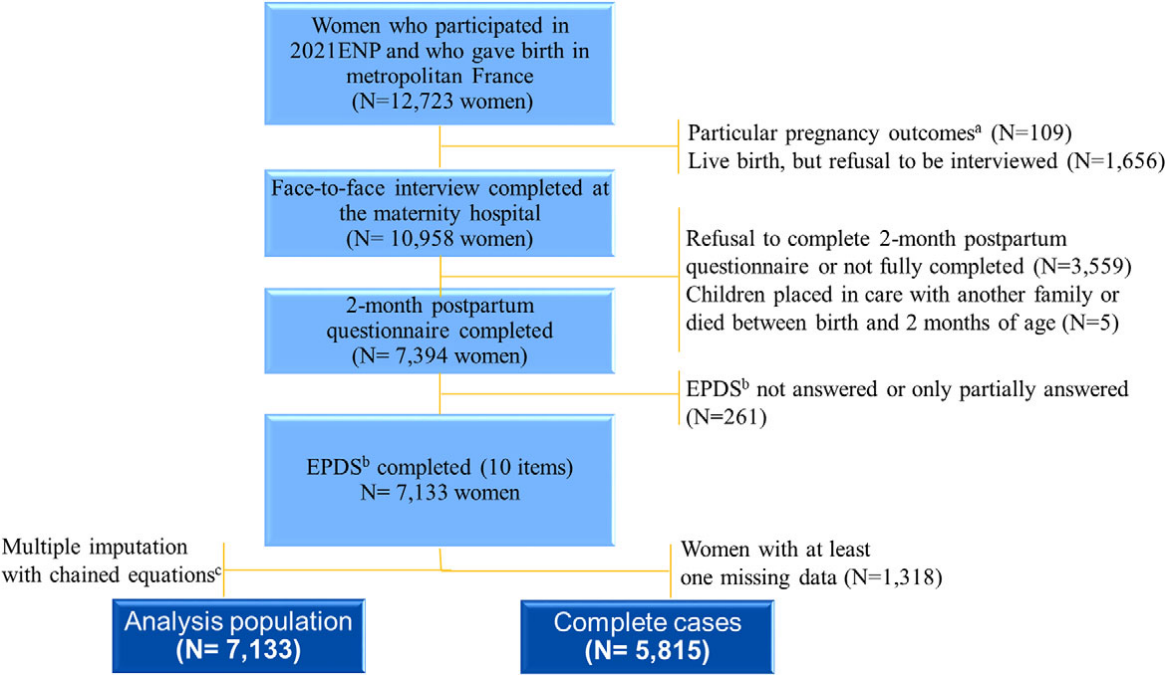
Flowchart of the study analysis, The 2021 French National Perinatal (2021 ENP) survey, France. a:Stillbirths, medical termination of pregnancy and secret births b:Edinburgh Postpartum Depression Scale (EPDS) c:Mechanism of non-response was ‘missing at random’ or ‘missing not at random’ which justified the use of multiple imputation; auxiliary variables used to perform multiple imputation by chain reaction were: Monthly household income (in euros), analgesia and depression symptoms at two months postpartum (Score EPDS>=13).

Among the 12,723 women who gave birth to at least one child in metropolitan France and who participated in 2021 ENP, 7,133 completed the 10 items of the EPDS in the 2-month postpartum questionnaire (median of 61 days postpartum, IQR [58–66]) (Flowchart, Figure 1). Those (i) with particular pregnancy outcomes (stillbirths, medical termination of pregnancy [MTP], and secret births), (ii) with a live child which was placed in care with another family or who died before 2-month follow-up, and (iii) who did not (fully) complete the EPDS were all excluded from the present analysis.

### PPA symptoms

The EPDS is a 10-item self-administered questionnaire that can be used to measure both perinatal depression and anxiety symptoms [22, 23]. Each item scores from 0 to 3, for a total possible score of 30. Anxiety symptoms are measured by the three-item EPDS-3A subscale which assesses a mother’s anxiety symptoms over the previous week. The three items are as follows: “I have blamed myself unnecessarily when things went wrong,” “I have been anxious or worried for no good reason,” and “I have felt scared or panicky for no good reason” [22]. We considered that women with an EPDS-3A score ≥5/9 had PPA symptoms [24, 25].

### Covariables

Explanatory variables were divided into five main themes (see below); their different categories are detailed in Table 1.

**Table 1.** Description of all women included in the study sample and those suffering from postpartum anxiety^a^ (PPA) symptoms at 2 months (prevalence and crude prevalence ratio); the 2021 French National Perinatal survey, France (*n* = 7,133).

	n^b^	%^c^	[95% CI]^c^	PPA symptoms prevalence			Crude PPA symptoms prevalence ratio (Bivariable analyses)		
				n^b^	%^c^	95% CI^c^	PR^d^	95% CI^d^	p-value^d^
**Study sample**	7,133	100.0	–	1,946	27.6	[26.5–28.8]	–	–	–
**Demographic and socioeconomic characteristics**									
Age (in years)^e^									**0.000**
15–24	615	10.7	[9.8–11.6]	200	32.2	[28.2–36.3[	**1.47**	[**1.25–1.73**]	
25–29	1,994	28.6	[27.4–29.8]	615	31.4	[29.1–33.7]	**1.43**	[**1.27–1.62**]	
30–34	2,708	36.4	[35.1–37.6]	723	26.8	[24.9–28.6]	**1.22**	[**1.08–1.38**]	
35–39	1,443	19.2	[18.2–20.1]	322	22.0	[19.7–24.2]	1(ref)	–	
≥40	373	5.1	[4.6–5.6]	86	24.6	[19.9–29.3]	1.12	[0.91–1.39]	
Country of birth									0.126
France	5,967	80.3	[79.2–81.5]	1,589	27.0	[25.8–28.2]	1(ref)		
Other European	220	3.6	[3.0–4.1]	61	26.6	[20.1–33.1]	0.98	[0.77–1.26]	
North Africa	346	6.9	[6.1–7.6]	105	29.1	[23.9–34.2]	1.07	[0.89–1.29]	
Other African	355	5.9	[5.2–6.6]	118	32.6	[27.3–37.9]	**1.21**	[**1.02–1.43**]	
Other country	175	3.3	[2.7–3.9]	50	33.4	[23.8–42.9]	1.24	[0.93–1.66]	
Not living with a partner	279	4.9	[4.2–5.5]	87	31.5	[25.5–37.6]	1.15	[0.94–1.40]	0.162
Educational level									0.781
<Secondary school diploma	992	19.1	[17.8–20.3]	288	28.6	[25.3–31.9]	1.05	[0.92–1.19]	
Secondary school diploma, 1 or 2 years tertiary education	2,722	39.2	[38.0–40.5]	733	27.4	[25.7–29.2]	1.00	[0.92–1.09]	
≥3 years tertiary education	3,419	41.7	[40.4–42.9]	925	27.4	[25.8–28.9]	1(ref)		
Monthly household income (in euros)									0.072
<1500	726	14.3	[13.2–15.5]	224	30.4	[26.6–34.2]	**1.15**	[**1.00–1.32**]	
1500–2900	2,110	32.8	[31.4–34.1]	592	28.4	[26.3–30.6]	1.07	[0.98–1.18]	
≥3000	4,090	52.9	[51.5–54.2]	1,076	26.4	[25.0–27.8]	1(ref)		
Not professionally active during pregnancy	1,581	29.6	[28.2–30.9]	460	29.1	[26.5–31.7]	1.07	[0.97–1.19]	0.156
Health literacy^f^	7,100	4.5	+/− 0.6	1,938	4.4	+/− 0.6	**1.32**	[**1.22–1.41**]	**0.000**
**Medical history/History of mental health care since adolescence**									
History of medical termination of pregnancy (MTP)	111	1.5	[1.2–1.8]	40	36.3	[26.4–46.3]	**1.32**	[**1.00–1.74**]	**0.049**
No consultation before planning to become pregnant	4,137	61.8	[60.6–63.0]	1,157	28.3	[26.7–29.8]	1.06	[0.98–1.15]	0.159
Mental health care history since adolescence^g^									**0.000**
None	5,564	84.8	[83.8–85.7]	1,402	25.9	[24.6–27.1]	1(ref)		
Psychological care (at least 3 months)	699	9.6	[8.9–10.4]	244	35.8	[32.0–39.5]	**1.39**	[**1.23–1.55**]	
Psychiatric care (at least 3 months) and/or hospitalization for a psychiatric reason	390	5.6	[4.9–6.2]	156	40.8	[35.6–45.9]	**1.57**	[**1.37–1.81**]	
**Pregnancy**									
Nulliparity	3,172	41.5	[40.2–42.7]	978	31.4	[29.7–33.1]	**1.26**	[**1.16–1.36**]	**0.000**
Body Mass Index (BMI) (kg/m^2^) before pregnancy									0.228
<25.0	4,545	62.0	[60.7–63.3]	1,216	27.1	[25.7–28.5]	1(ref)		
25.0–29.9	1,570	23.4	[22.2–24.6]	454	29.6	[26.9–32.2]	1.10	[0.98–1.21]	
≥30	964	14.6	[13.6–15.4]	257	26.9	[23.8–29.9]	0.99	[0.87–1.12]	
Weight change during pregnancy (in kg)									**0.000**
<=0	207	3.3	[2.9–3.9]	77	38.4	[31.1–45.7]	**1.46**	[**1.19–1.78**]	
1 to 8	1,245	18.6	[17.6–19.7]	356	28.8	[26.0–31.6]	1.10	[0.98–1.23]	
9 to 15	3,699	51.1	[49.7–52.3]	957	26.2	[24.6–27.7]	1(ref)	–	
16 to 22	1,639	23.1	[22.0–24.2]	436	27.1	[24.6–29.5]	1.03	[0.92–1.15]	
≥23	273	3.9	[3.5–4.4]	93	35.2	[29.2–41.1]	**1.34**	[**1.12–1.61**]	
Pregnancy-related emergency or walk-in consultation(s)									**0.000**
0	3,565	49.5	[48.1–50.7]	878	25.0	[23.5–26.6]	1(ref)		
1	1,972	27.4	[26.2–28.6]	527	26.8	[24.6–28.9]	1.07	[0.96–1.18]	
2	856	12.2	[11.3–13.1]	269	31.8	[28.1–35.4]	**1.27**	[**1.11–1.44**]	
≥3	722	10.9	[10.1–11.7]	269	36.9	[33.1–40.7]	**1.47**	[**1.31–1.66**]	
Perceived support from loved ones									**0.000**
Little or None	560	9.1	[8.2–9.9]	196	36.6	[31.5–41.7]	**1.46**	[**1.25–1.69**]	
Good	1,929	27.5	[26.4–28.7]	577	30.5	[28.1–32.7]	**1.21**	[**1.11–1.33**]	
Very good	4,614	63.3	[62.1–64.6]	1,166	25.1	[23.8–26.4]	1(ref)		
Not having a pregnancy at low obstetrical risk^h^	1,932	28.8	[27.5–29.9]	547	29.2	[26.8–31.6]	1.08	[0.98–1.19]	0.103
Feeling (for at least 2 weeks during the pregnancy)									**0.000**
Sadness, despair, hopelessness	880	12.4	[11.5–13.2]	316	35.3	[31.9–38.7]	**1.65**	[**1.47–1.84**]	
Anhedonia	417	6.1	[5.4–6.7]	141	34.5	[29.5–39.3]	**1.60**	[**1.37–1.86**]	
Both	883	14.0	[13.0–15.0]	416	47.7	[43.7–51.6]	**2.22**	[**2.00–2.45**]	
Neither	4,938	67.5	[66.3–68.8]	1,068	21.5	[20.2–22.7]	1(ref)		
**Childbirth**									
Mode of delivery									0.054
Spontaneous vaginal	4,755	66.5	[65.3–67.7]	1,245	26.6	[25.2–27.9]	1(ref)		
Instrumental vaginal	952	12.7	[11.9–13.5]	290	30.9	[27.8–33.9]	**1.16**	[**1.04–1.30**]	
Scheduled caesarean	474	7.1	[6.4–7.7]	125	27.2	[22.8–31.7]	1.03	[0.86–1.22]	
Emergency caesarean before labor	197	3.1	[2.7–3.6]	58	31.3	[24.0–38.5]	1.18	[0.93–1.49]	
Emergency caesarean during labor	755	10.6	[9.8–11.4]	228	29.7	[26.2–33.2]	1.12	[0.98–1.27]	
Not at all/not very satisfied with pain management received during childbirth	589	9.4	[8.6–10.2]	190	32.2	[28.0–36.3]	**1.18**	[**1.03–1.36**]	**0.016**
Analgesia									0.684
None	941	12.9	[12.1–13.7]	242	26.1	[23.1–29.1]	1(ref)		
Combined epidural, spinal or peri-rachial anesthesia	6,057	85.3	[84.4–86.2]	1,672	27.9	[26.7–29.2]	1.07	[0.94–1.21]	
Parenteral analgesia	81	1.2	[0.9–1.5]	20	24.5	[14.6–34.4]	0.94	[0.62–1.43]	
Other	43	0.5	[0.4–0.7]	11	26.7	[12.8–40.6]	1.02	[0.60–1.74]	
**Child’s health**									
Prematurity^i^	405	6.8	[6.0–7.5]	130	33.1	[27.5–38.7]	**1.21**	[**1.02–1.44**]	**0.029**
Weight for gestational age^j^									0.149
Small for gestational age (SGA) infant	822	12.5	[11.6–13.4]	247	30.1	[26.6–33.6]	1.09	[0.96–1.23]	
Appropriate for gestational age (AGA) infant	5,530	76.8	[75.6–77.9]	1,504	27.6	[26.3–28.9]	1(ref)		
Large for gestational age (LGA) infant	738	10.7	[9.8–11.5]	182	25.1	[21.6–28.7]	0.92	[0.79–1.07]	
Child hospitalization(s) during the first 2 months after leaving the maternity ward^g^	450	7.2	[6.5–7.9]	143	32.6	[27.8–37.3]	**1.19**	[**1.02–1.39**]	**0.022**

a Based on EPDS-3A, a subscale of the three items (3, 4 and 5) from the Edinburgh Postnatal Depression Scale (EPDS). A threshold ≥5 out of 9 is used for detecting postpartum anxiety symptoms.

b Number of women responding.

c Column percentage and related Confidence Interval 95% (95% CI) for qualitative variables or mean with standard deviation (+/−SD) for quantitative variables (weighted and imputed data). Percentages are presented per column and per row for all women and women with PPA symptoms, respectively.

d Crude Prevalence Ratio (PR) of PPA symptoms at 2 months, related 95% CI and global p-value (Poisson regression with robust error variance, weighted and imputed data). Significant associations (p < 0.05) appear in bold.

e At childbirth. The reference corresponds to the age group with the lowest prevalence and a sufficient number of women.

f Domain six of the Health Literacy Questionnaire (HLQ) (“Ability to actively engage with healthcare providers”). The higher the score the lower the level of health literacy.

g Declared by the mother at 2 months postpartum.

h Based on consensual French and international recommendations by the French National Authority for Health.

i <37 weeks of gestation.

j Small (>10th percentile), Appropriate (10th–90th percentile), and Large (>90th percentile) defined according to EPOPé curves adjusted for gestational age and sex.

#### Demographic and socioeconomic characteristics

Mother’s age at childbirth, mother’s country of birth, living with a partner or not, education level, monthly household income (in euros), professionally active during pregnancy, health literacy level during pregnancy (score of the sixth domain of the Health Literacy Questionnaire (HLQ) called “Ability to actively engage with healthcare providers,” validated in French) [26, 27].

#### Medical history

History of MTP, mental health care history since adolescence, assessed using three items: (i) “consulting a psychologist for at least 3 months,” (ii) “consulting a psychiatrist for at least 3 months,” (iii) “hospitalization for a psychological or psychiatric reason.” Women who answered “yes” only to (i) were categorized as receiving “psychological care”; those who answered “yes” to (ii) and/or (iii) were categorized as receiving “psychiatric care and/or hospitalization for a psychological/psychiatric reason”; finally, those who answered “no” to all three items, or to (i) and (ii) without documenting (iii) were categorized as receiving “no mental health care history since adolescence.”

#### Pregnancy-related characteristics

Nulliparity (excluding current childbirth), body mass index before pregnancy (in kg/m^2^), weight change during pregnancy (i.e., difference in kg between self-reported pre- and post-pregnancy weight) in five categories from ≤0 to ≥23 kg, self-reported pregnancy-related emergency or walk-in consultation(s), perceived support from loved ones during pregnancy, not having a pregnancy at low obstetrical-risk according to French guidelines (i.e., not meeting the A, A1, and A2 criteria defined in the consensual recommendations of the French National Authority for Health [28]), self-reported negative emotions and/or anhedonia for at least 2 weeks during their pregnancy (“Have you ever felt sadness, despair, hopelessness?”; “Have you ever experienced a loss of interest in most things, such as hobbies, work, or activities that usually give you pleasure?”).

#### Childbirth-related characteristics

Mode of delivery (five categories, including instrumental vaginal delivery and emergency cesarean delivery before or during labor; see details in Table 1), analgesia (on the basis of data collected in mothers’ medical records according to the mode of delivery), and not at all/not very satisfied with the pain management received during childbirth. The latter variable was created by dichotomizing the answer to the following item in the maternity ward interview: “Overall, are you satisfied with the pain management received to relieve pain and/or to help you during the contractions, irrespective of the pain-relief method used (including epidural analgesia)?” Women who answered “not at all satisfied” or “not very satisfied” were categorized in the “yes” answer modality of this variable; those answering “quite satisfied” or “very satisfied” were categorized in the “no” modality.

#### Child’s health characteristics

Prematurity (<37 weeks of gestation), birth weight according to gestational age (small, appropriate, and large, defined according to EPOPé curves, adjusted for gestational age and sex [29]), and child hospitalization(s) during the first 2 months of life.

For the present analysis, the subgroup of women with an EPDS score <13/30 was considered not to have PPD symptoms [30].

### Statistical analysis

Data collected at 2 months postpartum were weighted to take into account total non-response (modeled using the homogeneous response group technique [31, 32]) and to ensure our sample was representative of all women giving birth in all French maternity wards in the same week of March 2021.

We used a robust variance Poisson regression model to estimate the crude and adjusted prevalence ratios (aPR) [33] of having PPA symptoms at 2 months. The final multivariable model included covariables chosen based on previous work in the literature, our hypotheses, and the results of our bivariable analyses. The linearity of associations between the continuous variables included in the model (i.e., health literacy score and BMI) and the prevalence of PPA symptoms was confirmed using fractional polynomials. The multicollinearity of the demographic and socioeconomic variables was assessed using the generalized variance inflation factor.

For variables with fewer than 15 missing data, we imputed the missing data by using the most frequent modality response to that variable, taking into account other related available information if necessary. We then conducted multiple imputations with chained equations (MICE) [34] to address missing data (18.5%; max = 8.3%; min = 0.1%; see details of missing values in Supplementary Table 1). Twenty data sets were created in this process which included all variables from our multivariable final model, as well as auxiliary variables known to be associated with the variables to impute (Figure 1 and Supplementary Table 2). To better understand the impact of the MICE imputation on our results, we present the results of the “complete case analysis,” that is to say, the results of the final multivariable model on the database before the MICE imputation (Figure 1 and Supplementary Table 2).

The final multivariable model was used to study associated risk factors for three groups of women as follows: (i) the whole study population (*n* = 7,133), (ii) women with no history of mental health care since adolescence (*n* = 5,564) (as mental health problems history is a major and well-known risk factor for PPA symptoms), and (iii) women with no PPD symptoms at 2 months (*n* = 6,012) (to provide factors associated uniquely with PPA).

A sensitivity analysis was performed to check the robustness of the multivariable model for each of the three groups, after the exclusion of possible early markers of PPA symptoms, specifically the variables “Perceived support from loved ones during pregnancy,” “Self-reported pregnancy-related emergency visits or walk-in consultations” and “Not at all/not very satisfied with pain management during childbirth” (Supplementary Table 3).

The numbers of women responding for each modality are presented in Table 1. All other results presented are weighted. Imputed estimates are presented everywhere (except in Supplementary Tables 1 and 2) with prevalence ratios (i.e., crude or aPR), 95% CI, and associated *p*-values. As per Zou, prevalence ratios were interpreted in the same way as relative risks [35].

All statistical analyses were performed using Stata software version 16/SE^®^ (Stata Corp., College Station, TX).

### Institutional and ethical approval

The 2021 ENP received ethical approval from the following structures: the French National Council of Statistical Information (14 October 2019), the Committee of Ethics and Scientists for Research, Studies and Evaluations (CESREES) (12 June 2020), the Committee for the Protection of Persons (CPP) (7 July 2020), the Label Committee (Visa n°2021 × 701SA, 23 November 2020), and the National Data Protection Authority (CNIL) (DR-2020-391, 31 December 2020). Mothers were informed in participating maternity wards about the purpose of 2021 ENP before their inclusion in the survey; participation was voluntary. Parents of women under 18 years old (0.1%) who agreed to participate were also informed of the study content.

## Results

### Description of the study sample

Table 1 summarizes the characteristics of the 7,133 women included in our present analysis.

The majority were born in France (80.3%), were between 25 and 34 years old (65.0%), and were living with a partner (95.1%). They had a high level of education (41.7% had ≥3 years of tertiary education), 52.9% declared a household monthly financial income of at least 3,000 euros, and 70.4% worked during pregnancy. Forty-two percent were nulliparous.

Fifteen percent (15.2%) declared that they had received psychological or psychiatric care since adolescence. The proportion of women with PPD symptoms at 2 months was 16.7% (95% CI [15.7–17.7]); the mean EPDS score in the whole study population was 6.9 (sd = 5.3).

### Prevalence of PPA symptoms at 2 months in France and associated factors

Among the 7,133 women studied, 27.6% (95% CI [26.5–28.8]) were considered to have PPA symptoms at 2 months (i.e., EPDS-3A score ≥5/9).

After adjustment, risk factors independently and significantly associated with having PPA symptoms at 2 months were divided into four groups (echoing four of the explanatory variable themes used, see above) as follows: (i) socio-demographic characteristics, (ii) medical or mental health care history, (iii) pregnancy-related variables, and (iv) childbirth-related variables (Table 2). Note that the fifth theme – child’s health factors (including prematurity, weight for gestational age, and child hospitalization(s) during the two first months after leaving the maternity ward) was not associated with a higher risk of PPA symptoms (Table 2). Two socio-demographic characteristics were associated with having PPA symptoms: being under 34 years of age (the highest aPR being obtained for women aged between 25 and 29 years: 1.38; 95% CI [1.22–1.58] vs. 35–39 years) and having lower health literacy (1.15 [1.07–1.23]). Medical or mental health care history included having a history of medical termination of pregnancy (1.32 [1.05–1.68]), or psychological (1.31 [1.17–1.47]) or psychiatric (1.42 [1.24–1.63]) care since adolescence. Pregnancy-related variables included being nulliparous (1.23 [1.12–1.35]), no weight gain or loss during pregnancy (1.29 [1.03–1.61] vs. a gain of 9–15 kg), excessive weight gain (≥23 kg) during pregnancy (1.20 [1.00–1.43] vs. a gain of 9–15 kg), reporting three or more pregnancy-related emergency visits or walk-in consultations (1.16 [1.03–1.31] vs. none), self-perceiving no/poor or good support from loved ones during pregnancy (1.16 [1.00–1.34] and 1.15 [1.05–1.26], respectively, vs. very good support) and having experienced sadness (1.52 [1.36–1.69]), anhedonia (1.48 [1.27–1.72]) or both (1.99 [1.79–2.21]) for at least 2 weeks during pregnancy. The childbirth-related variable “not at all/not very satisfied with pain management during childbirth” was associated with a higher prevalence of PPA symptoms (1.16 [1.01–1.32] vs. quite/very satisfied).

**Table 2.** Factors associated with postpartum anxiety^a^ (PPA) symptoms at 2 months; data from the 2021 French National Perinatal survey, France (n = 7,133).

	Postpartum anxiety^a^ symptoms at 2 months (Multivariate analysis)					
	All women (n = 7,133)		Women with no history of mental health care (n = 5,564)		Women with no PPD symptoms (score EPDS<13) (n = 6,012)	
	Adjusted PR [95% CI]^b^	p-value^b^	Adjusted PR [95% CI]^b^	p-value^b^	Adjusted PR [95% CI]^b^	p-value^b^
**Demographic and socioeconomic characteristics**						
Age (in years)^c^		**0.000**		**0.000**		**0.000**
15–24	**1.33 [1.12–1.58]**		**1.36 [1.10–1.68]**		**1.45 [1.11–1.89]**	
25–29	**1.38 [1.22–1.58]**		**1.43 [1.23–1.67]**		**1.61 [1.33–1.96]**	
30–34	**1.21 [1.08–1.37]**		**1.26 [1.09–1.46]**		**1.30 [1.08–1.57]**	
35–39	1 (ref)		1 (ref)		1 (ref)	
≥40	1.03 [0.84–1.27]		1.12 [0.87–1.43]		0.94 [0.66–1.36]	
Country of birth		0.719		0.820		0.464
France	1 (ref)		1 (ref)		1 (ref)	
Other European	0.97 [0.76–1.22]		0.96 [0.73–1.27]		1.06 [0.75–1.49]	
North Africa	1.12 [0.92–1.34]		1.07 [0.88–1.32]		0.97 [0.69–1.34]	
Other African	1.03 [0.86–1.22]		1.02 [0.83–1.24]		1.22 [0.92–1.62]	
Other country	1.12 [0.86–1.46]		1.15 [0.88–1.50]		1.30 [0.82–2.06]	
Not living with a partner	0.90 [0.74–1.09]	0.284	0.87 [0.68–1.11]	0.258	0.96 [0.70–1.30]	0.781
Educational level		0.365		0.513		0.442
< Secondary school diploma	0.94 [0.83–1.07]		0.92 [0.79–1.07]		0.95 [0.78–1.15]	
Secondary school diploma and 1 or 2 years tertiary education	0.94 [0.86–1.03]		0.95 [0.86–1.06]		0.91 [0.79–1.04]	
≥3 years tertiary education	1 (ref)		1 (ref)		1 (ref)	
Not professionally active during pregnancy	0.94 [0.85–1.05]	0.280	0.95 [0.84–1.07]	0.373	0.88 [0.75–1.03]	0.119
Health literacy^d^	**1.15 [1.07–1.23]**	**0.000**	**1.16 [1.06–1.25]**	**0.000**	**1.15 [1.02–1.28]**	**0.019**
**Medical history/History of mental health care since adolescence**						
History of medical termination of pregnancy (MTP)	**1.32 [1.05–1.68]**	**0.019**	**1.44 [1.10–1.90]**	**0.008**	1.45 [0.93–2.27]	0.104
No consultation before planning to become pregnant	1.06 [0.98–1.15]	0.153	1.05 [0.95–1.15]	0.330	1.01 [0.90–1.14]	0.825
History of mental health care since adolescence^e^		**0.000**		–		**0.000**
None	1 (ref)		–		1 (ref)	
Psychological care (at least 3 months)	**1.31 [1.17–1.47]**		–		**1.39 [1.16–1.66]**	
Psychiatric care (at least 3 months) and/or hospitalization for a psychiatric problem	**1.42 [1.24–1.63]**		–		**1.41 [1.12–1.79]**	
**Pregnancy**						
Nulliparity	**1.23 [1.12–1.35]**	**0.000**	**1.25 [1.12–1.39]**	**0.000**	**1.30 [1.13–1.49]**	**0.000**
Body Mass Index (BMI) (kg/m^2^) before pregnancy	1.00 [0.99–1.01]	0.796	1.00 [0.99–1.01]	0.797	0.99 [0.98–1.00]	0.473
Weight change during pregnancy (in kg)		**0.033**		0.066		0.391
≤0	**1.29 [1.03–1.61]**		**1.31 [1.01–1.71]**		1.35 [0.95–1.91]	
1–8	1.08 [0.96–1.20]		1.07 [0.94–1.20]		1.03 [0.86–1.23]	
9–15	1 (ref)		1 (ref)		1 (ref)	
16–22	0.99 [0.89–1.10]		1.01 [0.90–1.14]		0.99 [0.86–1.15]	
≥23	**1.20 [1.00–1.43]**		1.22 [0.99–1.50]		1.09 [0.80–1.48]	
Pregnancy-related emergency or walk-in consultation(s)		0.288		0.127		0.216
0	1 (ref)		1 (ref)		1 (ref)	
1	1.01 [0.92–1.11]		1.03 [0.92–1.16]		1.05 [0.90–1.22]	
2	1.10 [0.97–1.24]		1.16 [1.00–1.34]		1.18 [0.98–1.43]	
≥3	**1.16 [1.03–1.31]**		**1.20 [1.04–1.38]**		**1.39 [1.15–1.68]**	
Perceived support from loved ones		**0.004**		**0.002**		0.311
Little or none	**1.16 [1.00–1.34]**		**1.24 [1.05–1.47]**		0.90 [0.67–1.21]	
Good	**1.15 [1.05–1.26]**		**1.17 [1.06–1.30]**		1.08 [0.95–1.24]	
Very good	1 (ref)		1 (ref)		1 (ref)	
Not having a pregnancy at low-obstetrical risk^f^	0.98 [0.89–1.09]	0.777	0.99 [0.88–1.12]	0.937	0.90 [0.76–1.07]	0.229
Feeling (for at least 2 weeks during the pregnancy)		**0.000**		**0.000**		**0.000**
Sadness, despair, hopelessness	**1.52 [1.35–1.69]**		**1.49 [1.31–1.70]**		**1.45 [1.22–1.72]**	
Anhedonia	**1.48 [1.27–1.72]**		**1.52 [1.29–1.80]**		**1.44 [1.15–1.82]**	
Both	**1.99 [1.79–2.21]**		**2.02 [1.78–2.29]**		**1.75 [1.46–2.09]**	
Neither	1 (ref)		1 (ref)		1 (ref)	
**Childbirth**						
Mode of delivery		0.846		0.620		0.323
Spontaneous vaginal	1 (ref)		1 (ref)		1 (ref)	
Instrumental vaginal	1.04 [0.92–1.17]		1.10 [0.97–1.26]		0.99 [0.83–1.18]	
Scheduled caesarean	1.08 [0.92–1.28]		1.05 [0.86–1.30]		1.13 [0.87–1.47]	
Emergency caesarean before labor	0.98 [0.77–1.24]		0.95 [0.73–1.25]		0.74 [0.49–1.11]	
Emergency caesarean during labor	1.03 [0.91–1.18]		1.04 [0.89–1.21]		1.12 [0.92–1.36]	
Not at all/not very satisfied with pain management received during childbirth	**1.16 [1.01–1.32]**	**0.035**	1.13 [0.96–1.33]	0.133	**1.24 [1.02–1.52]**	**0.032**
**Child’s Health**						
Prematurity^g^	1.09 [0.91–1.33]	0.324	1.15 [0.93–1.42]	0.190	1.26 [0.95–1.67]	0.112
Weight for gestational age^h^		0.419		0.606		0.367
Small for gestational age (SGA) infant	1.03 [0.91–1.17]		1.02 [0.88–1.19]		0.92 [0.75–1.11]	
Appropriate for gestational age (AGA) infant	1 (ref)		1 (ref)		1 (ref)	
Large for gestational age (LGA) infant	0.92 [0.79–1.06]		0.92 [0.78–1.10]		0.88 [0.71–1.09]	
Child hospitalization(s) during the first 2 months after leaving maternity ward^e^	1.13 [0.97–1.31]	0.107	**1.19 [1.00–1.41]**	**0.045**	1.19 [0.94–1.50]	0.142

a Based on EPDS-3A, a subscale of the three items (3, 4 and 5) from the Edinburgh Postnatal Depression Scale (EPDS). A threshold ≥5 out of 9 is used for detecting PPA symptoms.

b aPR of PPA symptoms at 2 months, related 95% CI and global p-value (Poisson regression with robust error variance, weighted and imputed data). Significant associations (p < 0.05) appear in bold.

c At childbirth. The reference corresponds to the age group with the lowest prevalence and a sufficient number of women.

d Domain six of the Health Literacy Questionnaire (HLQ) (“Ability to actively engage with healthcare providers”). The higher the score the lower the level of health literacy.

e Declared by the mother at 2 months postpartum (see questions asked in Methods section).

f Based on consensual French and international recommendations from the French National Authority for Health.

g <37 weeks of gestation.

h Small (>10th percentile), Appropriate (10th–90th percentile), and Large (>90th percentile) defined according to EPOPé curves adjusted for gestational age and sex.

### Factors associated with PPA symptoms at 2 months among women with no history of mental health care since adolescence

Among women with no history of mental health care since adolescence, 25.8% (95% CI [24.6–27.1]) had PPA symptoms at 2 months.

The risk factors for PPA symptoms in this subgroup (*n* = 5,564) were similar to those for the whole sample, apart from excessive weight gain (≥23 kg) and not at all/not very satisfied with the pain management received during childbirth. Furthermore, child hospitalization in the 2 months after returning home was significantly associated with having PPA symptoms (Table 2).

### Factors associated with PPA symptoms at 2 months among women with no PPD symptoms

Among women with no PPD symptoms at 2 months, 16.5% (95% CI [15.5–17.5]) had PPA symptoms at 2 months.

The risk factors for PPA symptoms in this subgroup were similar to those for the whole sample, apart from having a history of medical termination of pregnancy, weight change during pregnancy, and poor/no perceived support from loved ones during pregnancy (Table 2).

### Sensitivity analyses

The associated factors remained the same in the sensitivity analyses performed for each of the three groups when the markers of potential anxiety symptoms during pregnancy or while in the maternity ward (i.e., “Perceived support from loved ones during pregnancy,” “Self-reported pregnancy-related emergency visits or walk-in consultations” and “not at all/not very satisfied with pain management received during childbirth”) were excluded from the multivariable model (Supplementary Table 3).

## Discussion

The 2021 ENP survey examined a representative sample of all women who gave birth in France during the same week in March 2021. Of these, 27.6% (95% CI [26.5–28.8]) presented PPA symptoms (EPDS-3A score ≥5/9) at 2 months. In our analysis, several factors were significantly and independently associated with a higher risk of PPA symptoms at 2 months, and can serve as warning signals enabling clinicians to anticipate the onset of PPA. These factors can be categorized as (i) socio-demographic (younger age, lower level of health literacy), (ii) related to medical history (medical termination of pregnancy, history of mental health care since adolescence), (iii) related to the course of the pregnancy (nulliparity, poor/no perceived support from loved ones, feelings of sadness and/or anhedonia, three or more emergency visits or walk-in consultations for a pregnancy-related reason, no weight gain, weight loss, excessive weight gain), and (iv) related to childbirth (not at all/not very satisfied with pain management).

The factors associated with PPA symptoms in women with no history of mental health care since adolescence and women with no PPD symptoms at 2 months were similar to those for the whole population, with the exception of no/poor perceived support from loved ones, which was associated with a higher risk of PPA symptoms in no mental health care subgroup but not in the subgroup with no PPD symptoms.

### PPA symptom prevalence

PPA symptom prevalence at 2 months was 27.6%, which is higher than that reported in the 22-study meta-analysis by Dennis et al. (prevalence of 14.9% for 5–12 weeks postpartum) of 19,158 women [3]. However, some of the PPA symptom data in the latter came from countries whose economic situation was not comparable to that of France. Moreover, unlike our study, some of the 22 studies were not representative or population-based. In addition, most of the studies included used the self-reporting tool State-Trait Anxiety Inventory (STAI) which may partially explain the difference between the prevalence observed in our study and that in the meta-analysis.

When comparing our findings to those of the only other French study to date that asked women (from a university hospital) to complete the EPDS-3A tool at 2 months postpartum [24], PPA symptom prevalence was similar: 36.1% in that study (performed before the SARS-CoV-2 pandemic) vs. 41.1% in ours (conducted during the pandemic) with the same threshold (≥4).

### Factors associated with PPA symptoms

Few multivariable studies to date have examined risk factors for PPA symptoms. The factors they highlighted include a history of mental health issues, stress, the lack of social or partner support, a negative childbirth experience (including prematurity), and child health problems in the weeks following birth [9, 11, 12, 15]. Our results align with these findings. Furthermore, to the best of our knowledge, our study is the first to show that nulliparity and young maternal age are associated with PPA symptoms at 2 months.

Three other potential risk factors emerged from our analyses: lower health literacy, a history of medical termination of pregnancy, and “extreme” weight variations during pregnancy (weight loss, no weight gain, or excessive weight gain). In our study, health literacy was evaluated as a woman’s ability to actively engage in dialogue with healthcare providers. Our results highlight the importance of the patient-healthcare professional relationship in medical monitoring (compliance with care, treatment, and health examinations), and of constructive dialogue with health professionals to be able to access reliable information, be reassured, and receive appropriate care. With regard to a history of medical termination of pregnancy, a recent study showed that pregnant women who had already undergone a medical termination for a fetal abnormality were more likely to suffer from anxiety symptoms during their pregnancy [36]. We hypothesize that women with a history of termination are weakened by this past experience and may still be anxious 2 months postpartum. With regard to weight variations during pregnancy as a risk factor for PPA symptoms, we found a U-shaped association. A systematic review of the literature by Nagl et al. found only two studies focusing on the possible association between excessive gestational weight gain and anxiety [37]. Both showed that excessive weight gain was not related to anxiety in the pregnant population. Nevertheless, the authors of that review highlighted the need for further investigations to draw solid conclusions. In this context, longitudinal studies are needed to explore the association between weight gain and perinatal anxiety symptoms. Accordingly, in our study, we cannot exclude the possibility that weight changes during pregnancy reflect symptoms of prenatal anxiety identified in the postpartum period.

In our study, the risk factors for the subgroup of women with no history of mental health care were mostly similar to those for the whole population. Differences between both may perhaps be partly explained by a potential lack of power for both of the variables concerned (excessive weight gain during pregnancy (≥23 kg) and satisfaction with the pain management received during childbirth (i.e., not at all/not very satisfied)). We can also hypothesize that women with no history of mental health care since adolescence were less likely to have trait anxiety or a history of anxiety and were therefore less likely to have gained a lot of weight during their pregnancy or to answer not at all satisfied/not very satisfied to the question on the pain management received during childbirth. Moreover, for this subgroup, child hospitalization during the first 2 months of life was also associated with PPA symptoms, which corroborates data highlighting negative experiences and difficulties with children during the postnatal period in the general population [11, 12, 15].

Another major finding of our study was that women with PPA symptoms but no PPD symptoms had similar risk factors to those of the total study population, with the exception of three factors: medical termination of pregnancy, weight variation during pregnancy, and perceived support from loved ones during the pregnancy. For the two first variables, the discrepancy observed is probably due to study power. For the latter, compared with the total study population, the reversal of the prevalence ratio obtained suggests that little/no perceived support was not associated with having only PPA symptoms, whereas it was associated with having both PPA with comorbid PPD symptoms.

### Strengths and limitations

The strengths of our study include the fact that it was based on all maternity units and birthing or centers in France. Accordingly, it was possible to obtain a representative sample with a high level of detail in a large number of geographical areas. Moreover, the quality of the data, and in particular data completeness, was optimized by recruiting and training midwife interviewers working in maternity units.

Our analysis also has limitations. First, external validity is limited by attrition; nevertheless, data weighting should have minimized this problem. Second, our work was based on the use of the EPDS-3A, a three-item subscale within the EPDS self-administered questionnaire which is not the gold standard for screening anxiety symptoms. However, the EPDS is the most frequently used instrument to assess perinatal depression and is generally well-accepted by women. Despite recent work by Smith-Nielsen et al. showing that EPDS-3A is effective in detecting clinical levels of anxiety, further analyses are needed to validate the clinical relevance of the EPDS-3A subscale [25]. Third, the EPDS was used to screen for both PPD and PPA symptoms. Further analyses using separate screening tools are needed to confirm these results. Fourth, the 2021 ENP survey was conducted during the third wave of the SARS-CoV-2 pandemic in France; this too may have led to an overestimation of PPA symptom prevalence; however, it would not have biased the associated risk factors. Fifth, women who did not answer or who only partially completed the EPDS were excluded from the present analysis, and this may have generated selection bias. However, as this was a relatively small percentage (3.5%), any bias would have been limited. Sixth, the data collected in 2021 ENP did not allow us to adjust for anxiety symptoms during pregnancy or during childbirth; such symptoms may foster the development of PPA symptoms. Seventh, there are several sets of growth curves available in France. The classification of newborns according to their birthweight for their gestational age could thus have been done using other growth curves but we chose EPOPé’s because they are more recent than the other most commonly used curves and were constructed using data from a cohort representative of the French population.

Our study estimated that more than a quarter of women who delivered in France in 2021 had PPA symptoms at 2 months. It identified profiles of women at higher risk of PPA symptoms in the overall population; these were mostly similar to those found in the two subgroups of women with no history of mental health care and with no PPD symptoms. This highlights the importance for clinicians to consider screening specifically for PPA symptoms, over and beyond screening for PPD symptoms. Some of the risk factors identified in our study could be used to propose targeted prevention of PPA symptoms in the general population of pregnant women. Communicating information to women about the various symptoms of psychological distress is a particularly important issue. Talking groups run by peer associations can be very useful in breaking down isolation, a recurring factor in depression and perinatal anxiety symptoms. The French government’s “1000 first days” project and the European PATH project are two innovative interventions for the prevention and detection of perinatal mental health problems [38, 39]. Moreover, compulsory antenatal and postnatal interviews enabling women to discuss their situation with a healthcare professional (e.g., concerning their potential medical, social, or psychological difficulties and needs) could help to identify PPA symptoms and associated risk factors.

## Supporting information

Doncarli et al. supplementary material Doncarli et al. supplementary material

## Data Availability

The data set contains individual data potentially identifying or sensitive patient information (childbirth’s data, age, parity, region of residence, chronic diseases …). It cannot therefore be shared publicly. However, researchers who meet the criteria for access to confidential data can request access to these data by writing to DATA-MAD@santepubliquefrance.fr.

## Supplementary material

The supplementary material for this article can be found at http://doi.org/10.1192/j.eurpsy.2024.1799.
