# Supplementary material for: Prevalence of anxiety symptoms and associated factors at 2 months postpartum, results from a 2021 French national prospective cohort study
Source: Eur Psychiatry. 2024 Dec 27;67(1):e89. doi: 10.1192/j.eurpsy.2024.1799 (PMC11733619; doi:10.1192/j.eurpsy.2024.1799)
Supplement: Doncarli et al. supplementary material [file S0924933824017991sup001.docx]

## Supplementary Table 1: Missing data in our whole study sample and in two subgroups (i.e., women with no history of mental health care since adolescence; women with no PPD symptoms); the 2021 French National Perinatal survey, France (n=7,133).

|  | **All women (n=7,133)** | **Women with no history of mental health care since adolescence (n=5,564)** | **Women with no PPD^a^ symptoms (n=6,012)** |
| --- | --- | --- | --- |
|  | %* | %* | %* |
| **Total missing data^**^** | 18.5 | 12.5 | 18.1 |
| **Demographic and socio-economic characteristics** |  |  |  |
| Age (in years)^1^ | No missing data | | |
| Mother’s country of birth | 1.0 | 0.9 | 1.0 |
| Not living with a partner | No missing data | | |
| Educational level | No missing data | | |
| Monthly household income (in euros) | 2.9 | 3.0 | 2.7 |
| Not professionally active during pregnancy | No missing data | | |
| Health literacy^2^ | 0.5 | 0.4 | 0.4 |
| **Medical history / History of mental health care since adolescence** | | |  |
| History of medical termination of pregnancy (MTP) | 0.2 | 0.2 | 0.2 |
| No consultation before planning to become pregnant | 0.1 | <0.1 | <0.1 |
| Mental health care history since adolescence^3^ | 6.7 | - | 6.6. |
| Postpartum depression symptoms at two months^4^ | No missing data | | |
| **Pregnancy** |  |  |  |
| Nulliparity | No missing data | | |
| Body Mass Index BMI (kg/m^2^) before pregnancy | 0.8 | 0.7 | 0.7 |
| Weight change during pregnancy (in kg) | 1.0 | 1.0 | 0.9 |
| Pregnancy-related emergency visit(s) or walk-in consultation(s) | 0.3 | 0.2 | 0.3 |
| Self-perceived support from loved ones. | 0.4 | 0.4 | 0.4 |
| Not having a pregnancy at low obstetrical risk^5^ | No missing data | | |
| Feelings of sadness, despair, hopelessness and/or anhedonia for at least two weeks during the pregnancy | 0.2 | 0.2 | 0.2 |
| **Childbirth** |  |  |  |
| Mode of delivery | No missing data | | |
| Not at all/not very satisfied with pain management received during childbirth | 8.3 | 8.3 | 8.3 |
| Analgesia | 0.2 | 0.1 | 0.2 |
| **Child’s Health** |  |  |  |
| Prematurity^6^ | No missing data | | |
| Weight for gestational age | 0.6 | 0.6 | 0.5 |
| Child hospitalisation(s) during the first two months after leaving the maternity ward^3^ | 7.0 | 0.9 | 6.7 |

a Score EPDS <13

* Calculated for the set of variables used in the imputation process

** Calculated for the set of variables used in the final model

1 At childbirth

2 Domain six of the Health Literacy Questionnaire (HLQ) (“Ability to actively engage with healthcare providers”)

3 Self-declared at two months postpartum (see questions asked in Methods section)

4 Score EPDS>=13

5 Based on consensual French and international recommendations from the French National Authority for Health

6 <37 weeks of gestation

## Supplementary Table 2: Factors associated with postpartum anxiety^a^ (PPA) symptoms at two months. Comparison between the results obtained before (i.e., complete case analysis) and after multiple imputation; the 2021 French National Perinatal survey, France (n=7,133).

|  | **Postpartum anxiety**^a^ **symptoms at two months**  **(Multivariate analysis)** | | | | | | | |
| --- | --- | --- | --- | --- | --- | --- | --- | --- |
|  | **Complete case (n=5,815)** | | | | **Multiple imputation^b^ (n=7,133)** | | | |
|  | Adjusted PR^*^ [95% CI]^*^ | p-value^*^ | SE^*^ | (SE/PR)*100^*^ | Adjusted PR^*^  [95% CI]^*^ | p-value^*^ | SE^*^ | (SE/PR)*100^*^ |
| **Demographic and socioeconomic characteristics** | | | | |  |  |  |  |
| Age (in years)^1^ |  | **0.000** |  |  |  | **0.000** |  |  |
| 15-24 | **1.30 [1.07-1.57]** |  | **0.13** | **10.00** | **1.33 [1.12-1.58]** |  | **0.12** | **9.02** |
| 25-29 | **1.34 [1.16-1.54]** |  | **0.09** | **6.72** | **1.38 [1.22-1.58]** |  | **0.09** | **6.52** |
| 30-34 | **1.18 [1.04-1.35]** |  | **0.08** | **6.78** | **1.21 [1.08-1.37]** |  | **0.07** | **5.79** |
| 35-39 | 1(ref) |  |  |  | 1(ref) |  |  |  |
| >=40 | 0.96 [0.75-1.23] |  | 0.12 | 12.50 | 1.03 [0.84-1.27] |  | 0.11 | 10.68 |
| Country of birth |  | 0.526 |  |  |  | 0.719 |  |  |
| France | 1(ref) |  | - |  | 1(ref) |  |  |  |
| Other European | 1.04 [0.82-1.32] |  | 0.13 | 12.50 | 0.97 [0.76-1.22] |  | 0.11 | 11.34 |
| North Africa | 1.16 [0.94-1.43] |  | 0.12 | 10.34 | 1.12 [0.92-1.34] |  | 0.11 | 9.82 |
| Other African | 1.06 [0.87-1.30] |  | 0.11 | 10.37 | 1.03 [0.86-1.22] |  | 0.09 | 8.74 |
| Other country | 1.18 [0.88-1.57] |  | 0.17 | 14.40 | 1.12 [0.86-1.46] |  | 0.15 | 13.39 |
| Not living with a partner | 0.89 [0.71-1.11] | 0.295 | 0.10 | 11.24 | 0.9 [0.74-1.09] | 0.284 | 0.09 | 10.00 |
| Educational level |  | 0.128 |  |  |  | 0.365 |  |  |
| < Secondary school diploma | 0.91 [0.79-1.05] |  | 0.07 | 7.69 | 0.94 [0.83-1.07] |  | 0.06 | 6.38 |
| Secondary school diploma, 1 or 2 years tertiary education | 0.9 [0.82-0.99] |  | 0.05 | 5.56 | 0.94 [0.86-1.03] |  | 0.04 | 4.26 |
| >= 3 years tertiary education | 1(ref) |  |  |  | 1(ref) |  |  |  |
| Not professionally active during pregnancy | 0.92 [0.82-1.03] | 0.158 | 0.05 | 5.43 | 0.94 [0.85-1.05] | 0.280 | 0.05 | 5.32 |
| Health literacy^2^ | **1.12 [1.04-1.20]** | **0.004** | **0.03** | **3.37** | **1.15 [1.07-1.23]** | **0.000** | **0.03** | **3.45** |
| **Medical history / History of mental health care since adolescence** | | | | | | | | |
| History of medical termination of pregnancy (MTP) | **1.37 [1.08-1.74]** | **0.010** | **0.17** | **12.41** | **1.32 [1.05-1.68]** | **0.019** | **0.16** | **12.12** |
| No consultation before planning to become pregnant | 1.08 [0.98-1.17] | 0.103 | 0.05 | 4.63 | 1.06 [0.98-1.15] | 0.153 | 0.04 | 3.77 |
| Mental health care history since adolescence^3^ |  | **0.000** |  |  |  | **0.000** |  |  |
| None | 1(ref) |  | - |  | 1(ref) |  |  |  |
| Psychological care (at least 3 months) | **1.32 [1.17-1.49]** |  | **0.08** | **6.06** | **1.31 [1.17-1.47]** |  | **0.08** | **6.11** |
| Psychiatric care (at least 3 months) and/or hospitalisation for a psychiatric-related problem | **1.42 [1.23-1.64]** |  | **0.10** | **7.04** | **1.42 [1.24-1.63]** |  | **0.10** | **7.04** |
| **Pregnancy** |  |  |  |  |  |  |  |  |
| Nulliparity | **1.18 [1.07-1.30]** | **0.000** | **0.06** | **5.08** | **1.23 [1.12-1.35]** | **0.000** | **0.06** | **4.88** |
| BMI (kg/m2) before pregnancy | 1 [0.99-1.01] | 0.808 | 0.00 | 0.00 | 1 [0.99-1.01] | 0.796 | 0.00 | 0.00 |
| Weight change during pregnancy (in kg) |  | 0.173 |  |  |  | **0.033** |  |  |
| <=0 | 1.25 [0.98-1.58] |  | 0.15 | 12.00 | **1.29 [1.03-1.61]** |  | **0.14** | **10.85** |
| 1 to 8 | 1.08 [0.96-1.22] |  | 0.07 | 6.48 | 1.08 [0.96-1.20] |  | 0.06 | 5.56 |
| 9 to 15 | 1(ref) |  |  |  | 1(ref) |  |  |  |
| 16 to 22 | 0.97 [0.87-1.09] |  | 0.05 | 5.15 | 0.99 [0.89-1.10] |  | 0.05 | 5.05 |
| >=23 | 1.13 [0.92-1.39] |  | 0.12 | 10.62 | **1.19 [1.00-1.43]** |  | **0.11** | **9.17** |
| Pregnancy related emergency visit(s) or walk-in consultation(s) |  | **0.015** |  |  |  | 0.288 |  |  |
| 0 | 1(ref) |  |  |  | 1(ref) |  |  |  |
| 1 | 0.99 [0.89-1.10] |  | 0.05 | 5.05 | 1.01 [0.92-1.11] |  | 0.05 | 4.95 |
| 2 | 1.14 [0.99-1.30] |  | 0.08 | 7.02 | 1.1 [0.97-1.24] |  | 0.07 | 6.36 |
| >=3 | **1.2 [1.05-1.37]** |  | **0.08** | **6.67** | **1.16 [1.03-1.31]** |  | **0.07** | **6.03** |
| Perceived support from loved ones |  | **0.009** |  |  |  | **0.004** |  |  |
| Little or None | **1.2 [1.03-1.39]** |  | **0.09** | **7.50** | **1.16 [1.00-1.34]** |  | **0.09** | **7.76** |
| Good | **1.13 [1.02-1.24]** |  | **0.06** | **5.31** | **1.15 [1.05-1.26]** |  | **0.05** | **4.35** |
| Very good | 1(ref) |  | - |  | 1(ref) |  |  |  |
| Not having a pregnancy at low-obstetrical risk^4^ | 0.98 [0.88-1.10] | 0.801 | 0.05 | 5.10 | 0.98 [0.89-1.09] | 0.777 | 0.05 | 5.10 |
| Feeling (for at least two weeks during the pregnancy) |  | **0.000** |  |  |  | **0.000** |  |  |
| Sadness, despair, hopelessness | **1.49 [1.32-1.68]** |  | **0.09** | **6.04** | **1.52 [1.35-1.69]** |  | **0.08** | **5.26** |
| Anhedonia | **1.49 [1.27-1.75]** |  | **0.12** | **8.05** | **1.48 [1.27-1.72]** |  | **0.11** | **7.43** |
| Both | **1.93 [1.72-2.16]** |  | **0.11** | **5.70** | **1.99 [1.79-2.21]** |  | **0.10** | **5.03** |
| Neither | 1(ref) |  | - |  | 1(ref) |  |  |  |
| **Childbirth** |  |  |  |  |  |  |  |  |
| Mode of delivery |  | 0.566 |  |  |  | 0.846 |  |  |
| Spontaneous vaginal | 1(ref) |  | - |  | 1(ref) |  |  |  |
| Instrumental extraction | 1.06 [0.94-1.19] |  | 0.07 | 6.60 | 1.04 [0.92-1.17] |  | 0.06 | 5.77 |
| Scheduled caesarean | 1.07 [0.82-1.39] |  | 0.14 | 13.08 | 1.08 [0.92-1.28] |  | 0.09 | 8.33 |
| Emergency caesarean before labour | 1.07 [0.75-1.53] |  | 0.19 | 17.76 | 0.98 [0.77-1.24] |  | 0.12 | 12.24 |
| Emergency caesarean during labour | 1.12 [0.97-1.29] |  | 0.08 | 7.14 | 1.03 [0.91-1.18] |  | 0.07 | 6.80 |
| Not at all/no very satisfied with pain management received during childbirth | 1.12 [0.97-1.29] | 0.108 | 0.08 | 7.14 | **1.16 [1.01-1.32]** | **0.035** | **0.08** | **6.90** |
| **Child’s health** |  |  |  |  |  |  |  |  |
| Prematurity^5^ | 1.09 [0.88-1.35] | 0.409 | 0.12 | 11.01 | 1.09 [0.91-1.33] | 0.324 | 0.10 | 9.17 |
| Weight for gestational age^6^ |  | 0.407 |  |  |  | 0.419 |  |  |
| Small for gestational age (SGA) infant | 1.04 [0.91-1.19] |  | 0.07 | 6.73 | 1.03 [0.91-1.17] |  | 0.07 | 6.80 |
| Appropriate for gestational age (AGA) infant | 1(ref) |  | - |  | 1(ref) |  |  |  |
| Large for gestational age (LGA) infant | 0.91 [0.79-1.07] |  | 0.07 | 7.69 | 0.92 [0.79-1.06] |  | 0.07 | 7.61 |
| Child hospitalisation(s) during the two first months after leaving maternity ward^3^ | **1.19 [1.02-1.38]** | **0.028** | **0.09** | **7.56** | 1.13 [0.97-1.31] | 0.107 | 0.08 | 7.08 |

a Based on EPDS-3A, a subscale of the three items (3,4 and 5) from the Edinburgh Postnatal Depression Scale (EPDS).
A threshold >= 5 out of 9 is used for detecting postpartum anxiety symptoms

b See Methods section; Mechanism of non-response was missing at random or missing non at random which justified the use of multiple imputation; auxiliary variables used to perform multiple imputation by chain reaction were : Household month financial income (in euros), Type of analgesia and postpartum depression symptoms (score EPDS>=13)

* Adjusted Prevalence Ratio (aPR) of postpartum anxiety symptoms at two months, related 95%CI, standard deviation (SE), variation coefficient ((SE/PR)*100) and global p-value (Poisson regression with robust error variance, weighted and non-imputed (complete case) or imputed data). Significant associations (p<0.05) appear in bold

1 At childbirth. The reference corresponds to the age group with the lowest prevalence and a sufficient number of women.

2 Domain six of the Health Literacy Questionnaire (HLQ) (“Ability to actively engage with healthcare providers”). The higher the score the lower the level of health literacy

3 Declared by the mother at two months postpartum

4 Based on consensual French and international recommendations from the French National Authority for Health

5 <37 weeks of gestation

6 Small (>10^th^ percentile), Appropriate ([10^th^ -90^th^ percentile]) and Large (>90^th^ percentile) defined according to EPOPé curves adjusted for gestational age and sex

## Supplementary Table 3: Sensitivity analyses excluding the three covariables^a^ which were potential early makers of antenatal anxiety symptoms during pregnancy and childbirth; the 2021 French National Perinatal survey, France (n=7,133)

|  | **Postpartum anxiety^b^ symptoms at two months**  **(Multivariate analysis)** | | | | | |
| --- | --- | --- | --- | --- | --- | --- |
|  | **All women (n=7,133)** | | **Women with no history of mental health care (n=5,564)** | | **Women with no PPD^c^ symptoms (n=6,012)** | |
|  | Adjusted PR*  [95% CI]^*^ | p-value^*^ | Adjusted PR*  [95% CI]^*^ | p-value^*^ | Adjusted PR*  [95% CI]^*^ | p-value^*^ |
| **Demographic and socioeconomic characteristics** | | |  |  |  |  |
| Age (in years)^1^ |  | **0.000** |  | **0.000** |  | **0.000** |
| 15-24 | **1.34** [**1.13-1.59]** |  | **1.37 [1.11-1.69]** |  | **1.53 [1.18-1.99]** |  |
| 25-29 | **1.38** [**1.21-1.56]** |  | **1.43 [1.22-1.66]** |  | **1.62 [1.34-1.97]** |  |
| 30-34 | **1.21** [**1.07-1.36]** |  | **1.26 [1.09-1.45]** |  | **1.30 [1.08-1.57]** |  |
| 35-39 | 1(ref) |  | 1(ref) |  | 1(ref) |  |
| >=40 | 1.04 [0.84-1.28] |  | 1.11 [0.87-1.43] |  | 0.95 [0.66-1.37] |  |
| Country of birth |  | 0.597 |  | 0.651 |  | 0.528 |
| France | 1(ref) |  | 1(ref) |  | 1(ref) |  |
| Other European | 0.98 [0.77-1.24] |  | 0.97 [0.73-1.28] |  | 1.05 [0.74-1.48] |  |
| North Africa | 1.13 [0.94-1.37] |  | 1.10 [0.89-1.35] |  | 0.95 [0.69-1.32] |  |
| Other African | 1.04 [0.88-1.24] |  | 1.04 [0.86-1.27] |  | 1.21 [0.92-1.60] |  |
| Other country | 1.15 [0.88-1.49] |  | 1.19 [0.91-1.57] |  | 1.27 [0.81-2.00] |  |
| Not living with a partner | 0.91 [0.75-1.10] | 0.340 | 0.89 [0.70-1.13] | 0.333 | 0.95 [0.70-1.30] | 0.783 |
| Educational level |  | 0.365 |  | 0.499 |  | 0.523 |
| < Secondary school diploma | 0.94 [0.83-1.07] |  | 0.92 [0.80-1.07] |  | 0.95 [0.78-1.16] |  |
| Secondary school diploma, 1 or 2 years tertiary education | 0.94 [0.86-1.03] |  | 0.95 [0.85-1.05] |  | 0.92 [0.80-1.06] |  |
| >= 3 years tertiary education | 1(ref) |  | 1(ref) |  | 1(ref) |  |
| Not professionally active during pregnancy | 0.96 [0.86-1.06] | 0.400 | 0.96 [0.85-1.09 | 0.535 | 0.88 [0.75-1.04] | 0.131 |
| Health literacy^2^ | **1.18** [**1.10-1.26]** | **0.000** | **1.19 [1.10-1.28]** | **0.000** | **1.17 [1.04-1.30]** | **0.006** |
| **Medical history / History of mental health care since adolescence** | | |  |  | |  |
| History of medical termination of pregnancy (MTP) | **1.33 [1.05-1.68]** | **0.016** | **1.46 [1.12-1.92]** | **0.006** | 1.48 [0.94-2.34] | 0.091 |
| No consultation before planning to become pregnant | 1.06 [0.97-1.15] | 0.174 | 1.00 [0.99-1.01] | 0.353 | 1.00 [0.87-1.13] | 0.985 |
| Mental health care histories since adolescence^3^ |  | **0.000** |  | ‘- |  | **0.000** |
| None | 1(ref) |  | ‘- |  | 1(ref) |  |
| Psychological care (at least 3 months) | **1.32 [1.18-1.48]** |  | ‘- |  | **1.39 [1.16-1.66]** |  |
| Psychiatric care (at least 3 months) and/or hospitalisation for a psychiatric reason | **1.45 [1.26-1.65]** |  | ‘- |  | **1.45 [1.14-1.83]** |  |
| **Pregnancy** |  |  |  |  |  |  |
| Nulliparity | **1.21 [1.11-1.33]** | **0.000** | **1.23 [1.10-1.37]** | **0.000** | **1.13 [1.12-1.47]** | **0.000** |
| BMI (kg/m2) before pregnancy | 0.99 [0.99-1.01] | 0.887 | 1.05 [0.95-1.15] | 0.652 | 0.99 [0.98-1.00] | 0.606 |
| Weight change during pregnancy (in kg) |  | **0.024** |  | 0.060 |  | 0.280 |
| <=0 | **1.31 [1.05-1.63]** |  | **1.32 [1.01-1.71]** |  | 1.39 [0.98-1.97] |  |
| 1 to 8 | 1.07 [0.96-1.20] |  | 1.06 [0.93-1.21] |  | 1.03 [0.86-1.23] |  |
| 9 to 15 | 1(ref) |  | 1(ref) |  | 1(ref) |  |
| 16 to 22 | 1.00 [0.90-1.11] |  | 1.02 [0.91-1.15] |  | 1.00 [0.87-1.16] |  |
| >=23 | **1.21 [1.01-1.44]** |  | 1.22 [0.99-1.50] |  | 1.11 [0.82-1.51] |  |
| Pregnancy-related emergency visit(s) or walk-in consultation(s) | ‘- | ‘- | ‘- | ‘- | ‘- | ‘- |
| Pperceived support from loved ones | ‘- | ‘- | ‘- | ‘- | ‘- | ‘- |
| Not having a pregnancy at low obstetrical risk^4^ | 1.00 [0.90-1.10] | 0.987 | 1.02 [0.90-1.14] | 0.767 | 0.92 [0.78-1.10] | 0.369 |
| Feelings (for at least two week during the pregnancy) |  | **0.000** |  | **0.000** |  | **0.000** |
| Sadness, despair, hopelessness | **1.55 [1.39-1.73]** |  | **1.55 [1.36-1.77]** |  | **1.48 [1.25-1.75]** |  |
| Anhedonia | **1.51 [1.29-1.76]** |  | **1.55 [1.31-1.84]** |  | **1.46 [1.16-1.85]** |  |
| Both | **2.07 [1.86-2.29]** |  | **2.12 [1.87-2.40]** |  | **1.80 [1.50-2.17]** |  |
| Neither | 1(ref) |  | 1(ref) |  | 1(ref) |  |
| **Childbirth** |  |  |  |  |  |  |
| Mode of delivery |  | 0.912 |  | 0.667 |  | 0.418 |
| Spontaneous vaginal | 1(ref) |  | 1(ref) |  | 1(ref) |  |
| Instrumental vaginal | 1.03 [0.92-1.16] |  | 1.10 [0.96-1.25] |  | 0.98 [0.82-1.17] |  |
| Scheduled caesarean | 1.07 [0.90-1.27] |  | 1.04 [0.85-1.28] |  | 1.11 [0.85-1.45] |  |
| Emergency caesarean before labour | 0.98 [0.77-1.25] |  | 0.94 [0.72-1.24] |  | 0.75 [0.50-1.13] |  |
| Emergency caesarean during labour | 1.03 [0.89-1.17] |  | 1.03 [0.88-1.21] |  | 1.10 [0.90-1.34] |  |
| Poor or no satisfaction with pain management during childbirth | ‘- | ‘- | ‘- | ‘- | ‘- | ‘- |
| **Child’s Health** |  |  |  |  |  |  |
| Prematurity^5^ | 1.11 [0.92-1.34] | 0.273 | 1.18 [0.95-1.45] | 0.131 | 1.26 [0.95-1.69] | 0.108 |
| Weight for gestational age^6^ |  | 0.415 |  | 0.611 |  | 0.376 |
| Small for gestational age (SGA) infant | 1.03 [0.91-1.17] |  | 1.02 [0.88-1.18] |  | 0.92 [0.76-1.12] |  |
| Appropriate for gestational age (AGA) infant | 1(ref) |  | 1(ref) |  | 1(ref) |  |
| Large for gestational age (LGA) infant | 0.91 [0.79-1.06] |  | 0.92 [0.78-1.09] |  | 0.87 [0.70-1.08] |  |
| Child hospitalisation(s) during the two first months after leaving maternity ward^3^ | 1.14 [0.98-1.32] | 0.080 | **1.20 [1.01-1.42]** | **0.032** | 1.22 [0.97-1.54] | 0.089 |

a “Perceived support from loved ones during pregnancy”, “Emergency visit or walk-in consultation for pregnancy”, “Not at all/not very satisfied with the pain management received during childbirth”

b Based on EPDS-3A, a subscale of the three items (3, 4 and 5) from the Edinburgh Postnatal Depression Scale (EPDS). A threshold >= 5 out of 9 indicates postpartum anxiety symptoms

c EPDS score <13

* Adjusted Prevalence Ratio (aPR) of postpartum anxiety symptoms at two months, related 95%CI and global p-value (Poisson regression with robust error variance, weighted and imputed data). Significant associations (p<0.05) appear in bold

1 At childbirth. The reference corresponds to the age group with the lowest prevalence and a sufficient number of women.

2 Domain six of the Health Literacy Questionnaire (HLQ) (“Ability to actively engage with healthcare providers”). The higher the score, the lower the level of health literacy

3 Declared by the mother at two months postpartum

4 Based on consensual French and international recommendations from the French National Authority for Health

5 <37 weeks of gestation

6 Small (>10^th^ percentile), Appropriate ([10^th^ -90^th^ percentile]) and Large (>90^th^ percentile) defined according to EPOPé curves adjusted for gestational age and sex
